# Influenza vaccine effectiveness in patients hospitalized with severe acute respiratory infection in Lithuania during the 2019–2020 influenza season: a test negative case – control study

**DOI:** 10.1186/s12985-023-02015-0

**Published:** 2023-04-12

**Authors:** Roberta Vaikutyte, Monika Kuliese, Aukse Mickiene, Ligita Jancoriene, Birute Zablockiene, Giedre Gefenaite, Study group

**Affiliations:** 1 Department of Infectious Diseases, Lithuanian University of Health Sciences, Baltijos Street 120, Kaunas, 47116 Lithuania; 2 Clinic of Infectious Diseases and Dermatovenerology, Institute of Clinical Medicine, Faculty of Medicine, Vilnius University, Santariskiu street 14, Vilnius, 08406 Lithuania; 3 Center of Infectious Diseases, Vilnius University Hospital Santaros Klinikos, Santariskiu Street 14, Vilnius, 08406 Lithuania; 4 Department of Health Sciences, Faculty of Medicine, Lund University, Box 157, Lund, 22100 Sweden

**Keywords:** Influenza, Vaccine effectiveness, Hospital surveillance, Test-negative design

## Abstract

**Background:**

Influenza is a contagious viral airborne disease that adds to the clinical and economic burden on the healthcare system. It could be prevented substantially by seasonal influenza vaccination. Seasonal influenza vaccine effectiveness (SIVE) varies a lot and should therefore be monitored. This report aims to update age-stratified SIVE estimates among patients hospitalized due to severe acute respiratory infection (SARI) during the 2019–2020 influenza season.

**Methods:**

We performed a test-negative case-control study between December 2019 and April 2020 influenza season. We estimated SIVE and its 95% confidence intervals (95% CI) with logistic regression as (1-odds ratio)*100%. The models were adjusted for covariates that changed the unadjusted SIVE by ≥ 10%.

**Results:**

Among 84 participants, 32 (38.1%) were influenza positive, mostly with A(H1N1)pdm09 (25 cases; 78.1%). SIVE against any influenza adjusted for age and heart disease was 39.2% (95% CI: -119.3%, 83.1%). Age-stratified point estimates adjusted for heart diseases indicated different SIVE, and were 64.0% (95% CI: -309.2%, 96.8%) and 21.6% (95% CI: -252.2%, 82.6%) for 18–64 and ≥ 65 year-old participants, respectively.

**Conclusions:**

The point estimates suggested low to moderate SIVE against any influenza among hospitalized 18-64-year-old SARI participants, while low estimates were found in the ≥ 65-year-old group. Although broad SIVE confidence intervals indicate a small sample size and therefore the results can serve only as indicatory, they are in line with the estimates reported by other studies during the 2019–2020 season.

## Introduction

Influenza is one of the most common, contagious viral airborne diseases that adds a significant burden on the healthcare system by leading to severe complications such as pneumonia, respiratory failure, requiring hospitalization and even death [1, 2]. Our previous study showed that 49% of hospitalizations due to SARI during the influenza season were positive for influenza [3]. While 48% and 69% of SARI patients were influenza positive in the two infectious diseases departments, as high as 18% and 29% of patients in geriatric and internal disease departments, respectively were positive as well. Such high influenza positivity in the latter departments dedicated to noncommunicable diseases is striking and points to that the influenza disease burden is underestimated.

Annual vaccination against influenza is recommended for everyone, and it is especially promoted among people with an increased risk of attracting influenza or suffering from its complications. Globally, influenza vaccination rates have been gradually increasing over the last decades [4], between 2004 and 2013 the total number of distributed seasonal influenza vaccine doses increased by 87% (from approximately 262 to 490 million). In Lithuania, everyone over 65 years old, people with underlying medical conditions, pregnant women and health care workers are eligible to get vaccinated free of charge as part of the national vaccination campaign. Between 2013 and 2014 and 2020–2021 influenza seasons vaccination rates among the risk groups in Lithuania more than doubled [5, 6], yet the coverage in the general population is less than 10%. Vaccine coverage by specific risk groups in Lithuania is currently not available. However, based on the reported absolute number of vaccinated individuals over 65 years old and the number of people aged over 65 in Lithuania in 2019, we estimated that the 2019–2020 influenza season vacination rate for this age group was 18,7% [7, 8].

Seasonal influenza vaccine effectiveness (SIVE) is known to vary across seasons [9, 10]. One of the reasons is a mismatch between the vaccine strains and the circulating influenza strains [11]. It is also possible that the reliance on egg passaging for vaccine production can render additional mutations during the manufacturing process and therefore diminish SIVE [11, 12]. Next, people recommended to receive annual influenza vaccination often have a hampered immune system due to immunosenescence along the process of ageing, underlying medical or immunocompromised conditions, leading to weaker and shorter immunity following vaccination [12]. Finally, the timing between vaccination and the encounter with the virus is also important [13, 14]. The highest VE is observed shortly after vaccination (i.e., within 14 days), followed by six to eleven percent monthly decline, and remaining greater than zero for at least five to six months post-vaccination. There is evidence that SIVE wanes during the course of a single season [14].

It is important to track changes in SIVE between the seasons to understand its determinants as well as to further guide influenza prevention policies and strategies locally and globally. For this reason, in Lithuania SIVE was monitored as part of the collaboration with the I-MOVE network (Influenza – Monitoring Vaccine Effectiveness in Europe; https://www.imoveflu.org/) [15–17]. SIVE studies conducted by our study site contribute to public health policy and practice (inter) nationally, currently monitoring both, influenza and COVID-19 vaccine effectiveness. The aim of the current study was to continue SIVE surveillance in Lithuania through assessment of SIVE during the 2019–2020 influenza season.

## Methods

### Study design

We performed a test-negative case-control study among patients hospitalized with SARI in Kaunas Hospital of the Lithuanian University of Health Sciences and Vilnius University Hospital Santaros Clinics between December, 2019 and April, 2020. Cases were influenza positive patients confirmed by reverse transcription polymerase chain reaction (RT-PCR), and controls were influenza negative. For more details about the study design see elsewhere [16, 17].

The inclusion criteria were:

- Being 18–64 years old and having at least one underlying medical condition (heart, lung, rheumatologic, kidney diseases, diabetes, cancer, stroke, immunodeficiency and solid organ recipients, anemia, dementia) or being ≥ 65 years old; and.
- Being hospitalized due to SARI with at least one of the systemic symptoms (fever, myalgia, malaise, headache) or deterioration of general condition or deterioration of functional status, and at least one of the respiratory symptoms (sore throat, cough or dyspnea) for more than 24 h; and.
- Being swabbed within ≤ 7 days after self-reported disease onset; and.
- Not having had laboratory confirmed SARS-CoV-2 disease previously.

### Statistical analysis

Demographic, clinical and lifestyle characteristics (see Table 1) were collected to control for potential confounding. The differences between the characteristics of cases and controls were tested by Chi-square, Fisher’s Exact (2-sided) and Mann-Whitney tests (considered statistically significant at alpha level of < 5%). SIVE was estimated by logistic regression. The analysis was adjusted for (a set of) potential confounders if upon adjustment one by one they changed SIVE estimate by ≥ 10%.

**Table 1:** Demographic and clinical characteristics of controls and influenza cases

		Influenza negative N = 52 (61.9%)	Influenza positive N = 32 (38.1%)	p-value
Sex	Female	30 (35.7)	16 (19.0)	0.49_a_
Age, median (1st ; 3rd quartile)		72.5 (62.5; 82.8)	66 (43.5; 80.5)	0.15_c_
Age ≥ 65 y. o.		36 (42.9)	17 (20.2)	0.14_a_
SIV in the current season		12 (14.3)	4 (4.8)	0.23_a_
Current smoker		11 (13.1)	5 (6.0)	0.53_a_
At least one underlying medical condition:		49 (58.3)	28 (33.3)	0.42_b_
Heart diseases		37 (44.1)	14 (16.7)	0.01_a_
Diabetes		11 (13.1)	5 (6.0)	0.53_a_
Cancer		12 (14.3)	6 (7.1)	0.64_a_
Hematologic cancer		1 (1.2)	2 (2.4)	0.56_b_
Lung diseases		12 (14.3)	4 (4.8)	0.23_a_
Renal diseases		10 (11.9)	3 (3.6)	0.35_b_
Dementia		6 (7.1)	1 (1.2)	0.24_b_
Cirrhosis		2 (2.4)	0 (0.0)	0.52_b_
Anemia		6 (7.1)	1 (1.2)	0.24_b_
Immunodeficiency and solid organ recipients		2 (2.4)	4 (4.8)	0.20_b_
Obesity (BMI ≥ 30)		16 (19.3)	8 (9.5)	0.57_a_

a - Chi-square test; b - Fisher’s Exact test (2-sided); c – Mann-Whitney test

SIV – seasonal influenza vaccination

## Results

Eighty-four participants were included in the study, of which 32 (38.1%) were influenza positive. The most common influenza subtype was A(H1N1)pdm09 (25 cases; 78.1%), followed by A(H3N2) (5;15.6%) and B/Victoria (1 case).

While the results were not statistically significant, compared to controls, influenza cases were less often vaccinated, more often male and about six years younger (see Table 1). Except for hematologic cancer and immunodeficiency, underlying medical conditions were more prevalent among the controls, with only heart disease reaching statistical significance.

In 2019–2020 season unadjusted SIVE against any influenza among all participants was 52.4% (95% CI: -63.0%, 86.1%), which upon adjustment for potential confounders (i.e. heart diseases and age) decreased to 39.2% (95% CI: -119.3%, 83.1%). Adjusted SIVE estimates against any influenza were 64.0% (95% CI: -309.2%, 96.8%) and 21.6% (95% CI: -252.2%, 82.6%) for 18–64 and ≥ 65 year-old participants, respectively (Table 2).

**Table 2:** Seasonal influenza vaccine effectiveness by age groups

	Unadjusted SIVE %	95% CI	p-value of the unadjusted model	Adjusted SIVE %	95% CI	p-value of the adjusted model
**Any age**	52.4	-63.0 to 86.1	0.237	39.2*	-119.3 to 83.1	0.447
**Age 18–64 years**	69.0	-236.4 to 97.2	0.335	64.0**	-309.2 to 96.8	0.410
**Age ≥ 65 years**	35.7	-176.1 to 85.0	0.552	21.6**	-252.2 to 82.6	0.751

*Adjusted for heart diseases, age

** Adjusted for heart diseases

## Discussion

This study aimed to estimate SIVE in SARI patients for 2019–2020 influenza season. We found low to moderate SIVE against influenza among hospitalized patients. The age stratified analysis pointed to lower SIVE in older (21.6%) than in younger (64.0%) participants.

The point estimates from our study were in line with those reported by the I-MOVE multicenter study during the 2019–2020 season, where SIVE for all ages was found to vary between 29% and 61% against any influenza subtype, with somewhat lower estimates in older adults [18]. Studies from the United States and India also reported higher SIVE in younger than in older adults [19], which was possibly explained by the circulation of influenza B subtypes and the vaccine mismatch [20, 21].

According to the Lithuanian Compulsory Health Insurance Fund data, the incidence of clinically diagnosed influenza among adults had decreased from 21.5 cases per 1000 population in 2018, to 4.9 cases per 1000 population in 2020 [22, 23]. Similar decrease of influenza and other respiratory viral infections was reported in several other countries [24–26]. As compared to previous seasons, there were less SARI cases (SARS-CoV-2 cases excluded) requiring hospitalization during the 2019–2020 season both in Lithuania and worldwide [27]. While implementation of protective measures, such as wearing masks, washing and disinfecting hands more frequently, limiting travel and declaration of quarantine were likely contributors of such decrease [24], it might primarily be explained by the emergence and spread of SARS-CoV-2 at the beginning of 2020. Another reason for such decrease could be reduced influenza testing: as compared to previous season, the number of specimens tested with PCR in Lithuania during the 2019–2020 season reduced by 35% [28, 29]. During the 2020–2021 season, the number of laboratory-confirmed influenza cases in Lithuania dropped even further, with only 302 cases reported nationally [6]. Some cases of influenza could have also been masked by SARS-CoV-2 infection: studies reported influenza and SARS-CoV-2 coinfection to vary between 0.8 and 1.96%, and as high as 22.3% in severe patients [26, 30–32].

Several strengths and limitations of the current study should be mentioned. Due to a rather small sample size, and therefore broad confidence intervals, SIVE results of the current study should be interpreted with some caution. Stratification into different risks groups resulted in even smaller samples, limiting statistical power to explore further mechanisms leading to specific SIVE. As since the emergence of SARS-CoV-2 influenza testing was not performed, we were not able to include SARS-CoV-2 cases and so potential co-infections into the study and to estimate SARS-CoV-2 impact on SIVE.

One of the main strengths of the current study was using the generic protocol when it comes to the study design, data collection and analysis procedures [33], which makes the results easily comparable across the seasons and other studies that have adopted the protocol. Next, influenza confirmation was done by RT-PCR and the participants were recruited within seven days after SARI symptoms’ onset, which was expected to reduce the probability of false-negative results. Furthermore, cases were recruited before taking the swab and learning the results this way minimizing the classification bias.

## Conclusion

During the 2019–2020 season, which overlapped with the emergence of SARS-CoV-2 virus, there was a decline of influenza cases in Lithuania. The main circulating influenza subtype during the 2019–2020 season in Lithuania was A(H1N1)pdm09. The point estimates suggested low and moderate SIVE against any influenza among the older and younger hospitalized participants, respectively. Close monitoring of both influenza, SARS-CoV-2 and other respiratory pathogens incidence and co-infection data is an essential next step in better understanding SIVE estimates.

## Data Availability

The data presented in this study are available in insert article.

## Abbreviations

CI: confidence intervals
RT-PCR: Reverse transcription polymerase chain reaction
SARI: Severe acute respiratory infection
SIVE: Seasonal influenza vaccine effectiveness
